# Case Report: Disseminated Strongyloidiasis in a Patient with COVID-19

**DOI:** 10.4269/ajtmh.20-0699

**Published:** 2020-08-14

**Authors:** Audun J. Lier, Jessica J. Tuan, Matthew W. Davis, Nathan Paulson, Dayna McManus, Sheldon Campbell, David R. Peaper, Jeffrey E. Topal

**Affiliations:** 1 Section of Infectious Diseases, Department of Internal Medicine, Yale University School of Medicine, New Haven, Connecticut;; 2 Department of Pharmacy Services, Yale New Haven Hospital, New Haven, Connecticut;; 3 Department of Laboratory Medicine, Yale University School of Medicine, New Haven, Connecticut

## Abstract

The SARS-CoV-2 virus has emerged and rapidly evolved into a current global pandemic. Although bacterial and fungal coinfections have been associated with COVID-19, little is known about parasitic infection. We report a case of a COVID-19 patient who developed disseminated strongyloidiasis following treatment with high-dose corticosteroids and tocilizumab. Screening for *Strongyloides* infection should be pursued in individuals with COVID-19 who originate from endemic regions before initiating immunosuppressive therapy.

## INTRODUCTION

In December 2019, an outbreak of SARS CoV-2 emerged in Wuhan, China—rapidly evolving into a global pandemic of COVID-19. A single-center retrospective case series demonstrated bacterial and fungal coinfections among COVID-19–positive individuals.^1^ Further research is needed to investigate the relationship between COVID-19 and parasitic coinfection, particularly given the fact that patients with COVID-19 could receive immunosuppressive treatment, a potential risk factor for severe parasitic infection. This report describes the clinical features of a case of disseminated strongyloidiasis infection and polymicrobial bacteremia in an individual who received immunosuppressive treatment for COVID-19.

## CASE REPORT

A 68-year-old man presented to our institution with an 8-day history of chills, myalgia, headache, cough, nausea, and worsening dyspnea. The patient denied rhinorrhea, anosmia, dysgeusia, diarrhea, or abdominal pain.

Past medical history was significant for hypertension and diabetes mellitus complicated by peripheral neuropathy. Twenty years before, he had emigrated from Sucúa, Ecuador, and now resides in Connecticut. In Ecuador, he worked in a timber industry and also cultivated soil on farms.

On admission, temperature was 38.2°C, blood pressure 137/68 mm Hg, pulse 97 beats/minute, respiratory rate 24 breaths/minute, and oxygen saturation 96% on 2 L/minute of supplemental oxygen. On physical examination, he had dry mucous membranes and decreased air entry with bibasilar crackles. Admission blood work was notable for a white cell count of 10,200/mL^3^ (absolute lymphocyte count of 700/mL^3^ and absolute eosinophil count of 0/mL^3^), high-sensitivity C-reactive protein 178.8 mg/L, ferritin 885 mg/mL, D-dimer 1,200 ng/mL, hemoglobin A1c 6.5%. The admission chest X-ray was notable for bilateral patchy airspace opacities in the mid to lower lung zones. SARS-CoV-2 RNA was detected from a nasopharyngeal swab using the Cepheid Xpert Xpress SARS-CoV-2 assay. Sputum culture revealed normal commensal flora. He was admitted to the medicine unit and initiated on 5 days of hydroxychloroquine (400 mg oral twice daily loading dose and then 200 mg oral twice daily).

Following admission, he developed hypoxemic respiratory failure requiring intubation. Tocilizumab (once, intravenous at 8 mg/kg) was given as well as three courses of methylprednisolone (40 mg intravenous every 8 hours) on hospital days 4–6, 8–10, and 12–13 because of persistent hypoxemia and a new fever that manifested on day 9. On hospital day 12, he developed hypotension requiring norepinephrine for blood pressure support. *Streptococcus constellatus* and *Citrobacter freundii* were isolated on blood culture. Sputum culture from day 12 grew *Pseudomonas aeruginosa* and methicillin-susceptible *Staphylococcus aureus.* Methylprednisolone was discontinued, and he was initiated on intravenous ciprofloxacin, cefazolin, and metronidazole with resolution of fever and hypotension. Repeat blood cultures on days 14 and 16 revealed no growth.

**Figure 1.**
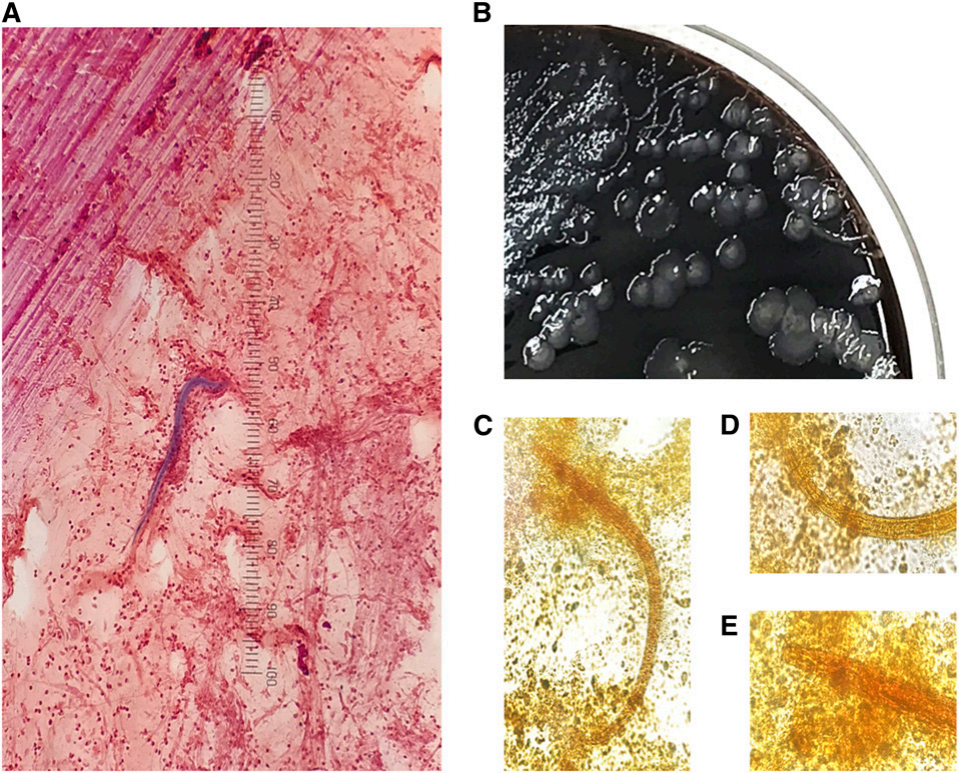
(**A**) First-stage rhabditiform larva (300 µm in length) of *Strongyloides* on sputum Gram stain. (**B**) Serpiginous tracks seen on the sputum culture chocolate agar plate. (**C**) *Strongyloides* larva (280 µm in length) on an iodine-stained wet mount of sputum with prominent genital primordium (**D**) and rhabditoid esophagus with short buccal canal (**E**). This figure appears in color at www.ajtmh.org.

On hospital day 18, he developed a new fever to 38.8°C while on the aforementioned antibiotic regimen without change to his ventilation settings. Absolute eosinophil count was 200/mL^3^. Antibiotics were changed to intravenous vancomycin and ciprofloxacin. His oxygenation improved, and he was extubated the following day. Sputum culture obtained on the same day grew *P. aeruginosa* and methicillin-sensitive *S. aureus*. The next day, serpiginous tracks were noted on a chocolate agar plate (Figure 1). Gram and iodine stains revealed larvae measuring 280–300 μm with a short buccal canal and prominent genital primordium consistent with *Strongyloides* species. The infectious diseases service was consulted and recommended initiation of ivermectin (200 μg/kg orally daily) for presumed strongyloidiasis and discontinuation of antibiotics.

On hospital day 21, his white cell count increased to 39,200/mL^3^ with an absolute eosinophil count of 100/mL^3^. Chest X-ray revealed unchanged multifocal bilateral pulmonary opacities. Given lack of significant clinical response to ivermectin, albendazole (400 mg orally every 12 hours) was added as adjunctive therapy. The evidence for this combination is low, but some efficacy has been seen in case reports.^2,3^ Piperacillin–tazobactam was initiated for suspected nosocomial pneumonia. In the ensuing days, his leukocytosis decreased. *Strongyloides* serum antibody and stool analysis for ova and parasites were negative. HIV and HTLV-1 serology were also negative.

On hospital day 25, he developed confusion, a new fever (39.4°C), hypotension requiring norepinephrine, and was subsequently reintubated. White cell count was 43,000/mL^3^. Blood cultures grew coagulase-negative *Staphylococcus* in one set*.* Repeat sputum culture grew *P. aeruginosa*, but no larvae were identified. A repeat stool ova and parasite was negative. Antibiotics were modified to intravenous vancomycin, ceftazidime, and metronidazole for possible bacterial meningitis associated with disseminated strongyloidiasis, in addition to other possible nosocomial infections. Because of hemodynamic instability, a lumbar puncture was deferred. In the ensuing days, his fever and hypotension resolved, mental status improved, and white cell count normalized. Notably, the absolute eosinophil count had peaked at 1,900/mL^3^ on day 28 and downtrended to 900/mL^3^ on day 33. A CT scan on hospital day 30 found widespread peripheral ground-glass opacities and peribronchial consolidation in the right lower lobe. Repeat blood and sputum cultures yielded no growth. A third stool ova and parasite was negative. He completed a 2-week course of ivermectin and albendazole as well as a 3-week antibiotic course for suspected Gram-negative meningitis. On day 38, repeat *Strongyloides* serology was positive. He currently awaits placement to a skilled nursing facility.

## DISCUSSION

Strongyloidiasis is caused by the soil-transmitted nematode *Strongyloides stercoralis*, which is endemic in tropical and subtropical regions with an estimated global prevalence of 30–100 million individuals.^4^ Our patient likely acquired the infection before emigrating to the United States from Ecuador in 2000. A recent study demonstrated that 20.7% of 1,418 serum samples from patients in eight provinces of Ecuador, one of which is near Sucúa, demonstrated positive antibody to *S. stercoralis*.^5^

*Strongyloides stercoralis* filariform larvae can penetrate the skin from the soil, enter the bloodstream, and subsequently migrate into the pulmonary vasculature, alveoli, and lungs. Via various mechanisms, larvae enter into the small intestine where they develop into adult female worms and reproduce by parthenogenesis.^4^ Unique to *S. stercoralis* among soil-transmitted helminths is that eggs hatch into rhabditiform larvae in the intestine, rather than in the environment, which can reinfect the human host by entering the intestinal wall or perianal skin without exogenous reinfestation.^4,6^ This unique process of autoinfection can yield lifelong, often asymptomatic or subclinical infection. In addition, strongyloidiasis has been associated with immunosuppressive diseases, causing cell-mediated immune deficits including HTLV-1 and HIV infection.^4,6^

One of the strongest risk factors associated with *Strongyloides* dissemination or hyperinfection syndrome has been the use of corticosteroids, likely secondary to inhibition of eosinophil and lymphocyte activation.^7^ In addition, corticosteroid use increases the fertility of adult female worms, leading to increased numbers of eggs and subsequent rhabditiform larvae.^8^ However, there does not appear to be a defined correlation between corticosteroid duration or frequency and development of strongyloidiasis.^7^ Disseminated strongyloidiasis occurs when larvae invade end organs not classically associated with the normal life cycle of the parasite. Gram-negative sepsis and meningitis are attributed to *Strongyloides* larvae assisting enteric bacteria with entering the host’s bloodstream via the gut mucosa.^9^

Our case had several important clinical features. First, in addition to receiving the anti-IL-6 receptor antibody tocilizumab, the patient also received three courses of high-dose methylprednisolone to mitigate possible COVID-19–related cytokine release syndrome.^10^ The most likely predisposing risk factor was receipt of corticosteroids, as tocilizumab alone has not been linked to the development of disseminated strongyloidiasis. A single case report describing disseminated strongyloidiasis after receipt of tocilizumab was in the context of concomitant corticosteroid use (T. T. Maffort, unpublished data).

A second important clinical consideration is the importance of early suspicion for disseminated strongyloidiasis when associated with Gram-negative bacteremia and signs of meningitis. A retrospective study of disseminated strongyloidiasis demonstrated that 23 of 70 cases were associated with sepsis due to enteric organisms. Furthermore, 12 of 70 cases had manifestations of meningitis, most frequently with Gram-negative organisms, whereas 26 of 70 cases had suppurative culture-negative meningitis.^11^ Given our patient’s epidemiologic risk factor and development of Gram-negative bacteremia, the clinical diagnosis of disseminated strongyloidiasis was further supported.

Finally, our case illustrates the challenge with performing diagnostic testing in immunosuppressed individuals. The initial *Strongyloides* serology on hospital day 21 was negative, but the repeat was positive on day 38. The *Strongyloides* IgG serology for the L3 antigen by ELISA (New Life Diagnostics, Carlsbad, CA; performed by Quest Diagnostics, San Juan Capistrano, CA) has a 92% sensitivity and 100% specificity.^12^ Consequently, the initial negative serology was likely attributable to the net state of immunosuppression following receipt of multiple courses of high-dose corticosteroids, which has been previously described.^13^ In addition, serial stool ova and parasite testing was negative, which may be due to the limited sensitivity of stool testing in the setting of disseminated strongyloidiasis.^13^ However, the discovery of tracks of bacterial colonies in sputum culture with identification of rhabditiform larvae with defining morphologic characteristics (appropriate size, short buccal canal, and prominent genital primordium) provided a definitive diagnosis in the clinical context.

## CONCLUSION

The COVID-19 pandemic has prompted important discussions over its optimal management with the role of corticosteroids and tocilizumab yet to be fully defined.^8^ This case highlights important considerations when using immunosuppressive therapies for COVID-19 treatment, particularly in patients with risk factors for prior *Strongyloides* infection. Clinical suspicion for disseminated strongyloidiasis should be maintained in patients from endemic areas who develop Gram-negative sepsis or meningitis. Screening is challenging in this clinical setting, given the rapidity with which COVID-19 can progress. Therefore, before administering immunosuppressive therapy in COVID-19 patients, a structured screening mechanism and implementation of a definitive approach may be warranted. We suggest performing *Strongyloides* serology in patients from endemic regions before receiving an immunosuppressive treatment regimen. Once the *Strongyloides* serologic status is known, perhaps additional screening with HTLV-1 testing and stool analysis could be pursued.
